# COVID-19 and the craniomaxillofacial surgical community

**DOI:** 10.1177/1943387520921325

**Published:** 2020-04-16

**Authors:** Srinivas M. Susarla, Sat Parmar, Rui Fernandes

The global outbreak of the novel coronavirus, SARS-CoV-2, or COVID-19, has dramatically transformed all aspects of our daily lives. The continually evolving understanding of the disease process and uncertainty regarding the availability of vital resources have created an unprecedented challenge, matched in magnitude by concerns for the well-being of our patients, families, colleagues, and communities. Our global craniomaxillofacial (CMF) community has met this challenge head on, whether providing care for acute injuries or aggressive pathology, performing modified duties in support of our frontline medical colleagues, assisting in the development of triage and care plans, supporting public health responses, or assisting friends and neighbors in this austere climate.

Public health measures for containing the spread of disease (social distancing) and to optimize the allocation of limited resources, such as personal protective equipment, have now been widely adopted and there is hope that they will be effective. Similarly, principles for the safe practice of craniomaxillofacial surgery during this crisis cannot be overemphasized. The AO CMF International Task Force has developed guidelines to support surgeons caring for patients during the COVID-19 pandemic.^1^ These recommendations trace their evolution to expert opinions and guidelines set forth by a number of national and international societies, as well as the limited, but increasing, available data in the peer-reviewed medical literature. The complete report from the Task Force is available online, and we strongly encourage you to review this document, if you have not already had an opportunity to do so.^1^ The Executive Summary, highlighting the critical recommendations, emphasizes the following:

- Surgical procedures involving the nasal–oral mucosal regions are high risk for infection of medical personnel due to aerosolization of the COVID-19 virus.
- Asymptomatic patients may be infected with COVID-19 virus.
- Elective procedures and routine ambulatory visits should be canceled.
- Appropriate PPE should be worn during surgical procedures and urgent ambulatory visits, which includes N95/full face shield or Powered Air Purifying Respirator (PAPR).
- Intraoperative measures that limit the generation of aerosolized virus are recommended.
- Oncologic cases in which a worse outcome is expected if surgery is delayed more than 6 weeks should be performed with appropriate Personal Protective Equipment (PPE).

Due to the precipitous dynamics of this crisis, these recommendations may change in the coming weeks to months. We encourage you to continue to remain involved with your local and regional health authorities, contribute to the active dialogues regarding provision of craniomaxillofacial surgical services, and provide clinical care where appropriate.

Thank you all for your continued fortitude and flexibility in support of your local and regional communities. Please stay safe and be well.

Srinivas M. Susarla, Sat Parmar, Rui Fernandes
